# Comparison of Fungal Genera Isolated from Cucumber Plants and Rhizosphere Soil by Using Various Cultural Media

**DOI:** 10.3390/jof9090934

**Published:** 2023-09-15

**Authors:** Chong-Yang Cheng, Ming-Yuan Zhang, Yong-Chun Niu, Meng Zhang, Yue-Hua Geng, Hui Deng

**Affiliations:** 1 Plant Protection College, Henan Agricultural University, No. 95 Wen-Hua Road, Zhengzhou 450002, China; 15138557986@163.com (C.-Y.C.); zm2006@126.com (M.Z.); 2 State Key Laboratory of Efficient Utilization of Arid and Semi-arid Arable Land in Northern China, Institute of Agricultural Resources and Regional Planning, Chinese Academy of Agricultural Sciences, No. 12 Zhongguancun South Street, Beijing 100081, China; 13012617396@163.com (M.-Y.Z.); niuyongchun@aliyun.com (Y.-C.N.); 3 Key Laboratory of Microbial Resources Collection and Preservation, Ministry of Agriculture and Rural Affairs, Institute of Agricultural Resources and Regional Planning, Chinese Academy of Agricultural Sciences, No. 12 Zhongguancun South Street, Beijing 100081, China

**Keywords:** plant endophytic fungi, rhizospheric soil fungi, isolation, *Cucumis sativus* L., cultural media

## Abstract

Plant endophytic fungi and rhizosphere soil fungi are often reported as biocontrol agents against plant pathogens or with plant growth promotion potential. Four treatments were performed in field and greenhouse experiments where cucumber plants were inoculated with *Trichoderma harzianum* and *Fusarium oxysporum* in 2022. The roots, stems and leaves of cucumber plants and their rhizosphere soil were collected twice individually from the field and greenhouse for isolation of cucumber endophytic and rhizosphere soil fungi. All fungal strains were identified through sequence similarity of the ITS1-5.8s-ITS2 rDNA region. The potato dextrose agar (PDA) media yielded the highest number of genera isolated from cucumber plants, rhizosphere soil and both compared to other media. There were no significant differences among the four media for the isolation of all cucumber endophytic fungi. However, in the roots, the number of endophytic fungi isolated by MRBA was significantly higher than that isolated on malt extract agar (MEA), while in the stems, the number of fungi isolated with PDA was significantly higher than that isolated with Martin’s rose bengal agar medium (MRBA). PDA had significantly higher isolation efficiency for the rhizosphere soil fungi than MRBA. The 28 fungal genera had high isolation efficiency, and the endophytic *Trichoderma* strains were significantly more isolated by MEA than those of MRBA. It is suggested that PDA can be used as a basic medium, and different cultural media can be considered for specific fungal genera.

## 1. Introduction

Fungi occurring in host plants as endophytes have been demonstrated to act as biocontrol agents against plant pathogens and have plant growth-promoting potential [1]. Some soil fungi have been widely studied and commercially marketed as biopesticides, biofertilizers and soil amendments [2]. There are several reports of different isolation media being used to isolate diverse culturable fungi from plants and soil [3,4].

Different media are an important factor affecting the isolation of endophytic fungi [5,6,7]. Culturable endophytic fungal species from *Artemisia Tuscola* have a “preference” for nutrient medium, and using different culture media to obtain a higher diversity of species was suggested [8]. Many studies used different media for the isolation of fungi, and most researchers know that using a wide variety of media is the best way to obtain higher fungal diversity [4,5,6,7,8,9,10,11,12,13,14,15,16,17,18,19,20,21,22]. There are various media available for the isolation of plant endophytic fungi, in which PDA is the most common medium [4,5,7,9,12,14,15,18,20,21,22], and PDA has been reported to have better isolation efficacy than other media [5,22]. Malt extract agar (MEA) is the second most commonly used material to isolate endophytic fungi from plants [4,5,12]. For isolating endophytic fungi, rose bengal agar medium (RBA) [19] and corn meal agar (CMA) [11,13] were also chosen less often than PDA and MEA, while other media, such as Czapek agar medium (CZA) [18,22], Hagem agar medium [18], oat meal agar (OA) [12] and V8 juice medium, were rarely used [4].

To isolate as many soil fungi as possible, one to four different media [23,24,25,26,27,28,29,30] are often applied for isolation. PDA [24,26,30], CZA [26,31,32], RBA [27,33], Martin’s rose bengal agar medium (MRBA) [25] and MEA [23,29,30,32] are common media for isolating soil fungi. Other media, such as Martin medium [24,30], water agar (WA) [26,32] and YM [23], are also used. Among PDA, CZA and WA, PDA resulted in a large number of fungal strains, and most strains were common to both PDA and CZA, while WA supported pycnidia-producing fungi [26]. When Martin medium was used, the number of total fungi in sorghum, eucalyptus and forested soils showed an increase of 1.3- to 1.7-fold higher than that in CZA medium [31].

Plant endophytic fungi and soil fungi have abundant populations, and the selection of suitable medium or different media is one of the key factors for successful isolation of fungal diversity. In this study, to isolate both plant endophytic fungi and soil fungi as fully as possible, a combination of multiple media was used to increase the effectiveness of fungal isolation. During two growth periods of cucumber in the field and greenhouse, potato dextrose medium (PDA), malt extract medium (MEA), Martin’s rose bengal agar medium (MRBA) and corn powder agar medium (CMA) were selected to isolate endophytic fungi from cucumber plants, and Czapek agar medium (CZA), PDA and MRBA were used to isolate soil fungi from the cucumber rhizosphere. A total of five different media were tested to optimize isolation media to isolate various communities of cucumber plants and rhizosphere soils.

## 2. Materials and Methods

### 2.1. Trial Design for Plants and Soil Sampling

Starting from 20 June 2022 and 12 July 2022, the healthy plants of *Cucumis sativus* L. variety “Jinyan No. 2”, which was susceptible to cucumber disease of *Fusarium oxysporum* f. sp. *cucumerinum*, were separately grown in the greenhouse and the field at the Chinese Academy of Agricultural Sciences (39°57′34″ N, 116°19′19″ E), for study the plant endophytic and rhizosphere fungi. The cucumber plants were placed in the greenhouse at 25 °C/18 °C (light/dark) with a 15 h photoperiod per day. In previous seasons, the healthy *Zea maydis* L., *Nicotiana tabacum* L. and cucumber were once grown in the greenhouse, while healthy *Triticum aestivum* L. and cucumber were rotated in the field.

The four treatments conducted in this study were similar to a previous study of the potential of endophytic fungi for biocontrol of soilborne fungal diseases of cucumber [34]: control (CC), inoculation with *Trichoderma harzianum* (CT), inoculation with *Fusarium oxysporum* f. sp. *cucumerinum* (CF) and inoculation with *T. harzianum* and *F. oxysporum* f. sp. *cucumerinum* (TF). The strain *Trichoderma harzianum* (strain no. F30) was isolated and preserved in our lab with biocontrol potential of cucurbit pathogenic fungi *Fusarium oxysporum* f. sp. *cucumerinum* (strain no. ACCC 37438, preserved in the Agricultural Culture Collection of China). Preparation of endophytic fungal inoculum, sowing of cucumber and inoculation of endophytic fungi and preparation of pathogenic inoculum and artificial inoculation followed our previous work [34]; the final inoculation of *T. harzianum* is 1 g of endophytic fungal inoculum per plant and 10 mL of 10^7^ spores per ml pathogenic inoculum per plant. The field experiment was carried out in a field with length × width = 15 m × 1.2 m and 20 rows, and each treatment consisted of 5 rows. There were 10 cucumber plants in each row, for a total of 200 cucumber plants. Samples were collected from the field and greenhouse twice separately (Table 1), and in the field, each plant sample was collected from one row for each treatment. The soil was collected along with the whole plant, wrapped in a sterilized paper bag and immediately transported to the laboratory. In the greenhouse experiment, each treatment contained 30 plants, and a total of 120 plants were grown. One cucumber plant was grown in each sterile pot (diameter × height = 10 cm×10 cm) filled with 500 g of the soil from the field. Samples were collected twice from the greenhouse experiments (Table 1). The fungi were isolated from the roots, stems and leaves of cucumber plants 48 h [35] after collection [36]. The soil was stored in sterilized polythene bags at 4 °C and isolated within 7 days [37].

### 2.2. Cultural Media

Four different cultural media were used for isolation of cucumber endophytic fungi: CMA [36] (corn meal 2 g, agar 15 g, distilled water 1000 mL, Becton, Dickinson & Co., Sparks, MD, USA), MEA [39] (malt extract 30 g, mycological peptone 5 g, agar 15 g, distilled water 1000 mL, Oxoid Ltd., Basingstoke, Hampshire, England, UK), MRBA [25] (mycologica peptone 5.00 g, D-glucose 10.00 g, potassium phosphate 1.00 g, magnesium sulfate 0.50 g, rose bengal 0.03 g, chloramphenicol 0.10 g, agar 15.00 g, distilled water 1000 mL) and potato dextrose agar (PDA) [40] (potato 200 g, D-glucose 20 g, agar 20 g, distilled water 1000 mL), while three media, CZA (NaNO_3_ 2.00 g, K_2_HPO_4_ 1.00 g, KCl 0.50 g, MgSO_4_·7H_2_O 0.50 g, FeSO_4_·7H_2_O 0.01 g, D-saccharose 30.00 g, agar 15.00 g, distilled water 1000 mL), MRBA and PDA were used for isolation of rhizosphere soil fungi. Except for MRBA, ampicillin and chloramphenicol were added to the other four culture media so that the concentration of ampicillin and chloramphenicol reached 10 μg/mL, which is a modification of the concentration used by Fang (1998) [41], to inhibit the growth of bacteria.

### 2.3. Isolation of Endophytic and Rhizosphere Soil Fungi

The plant tissues were cut into 1–2 cm blocks [39], soaked in 70% ethanol for 30 s, transferred to 4% NaClO, immersed for 2 min and rinsed with sterile water 3 times, and the surface moisture was absorbed by sterile filter paper [36]. Drops of sterilization water from the last step were poured on medium plates as a control check for complete sterilization. After the dried tissue blocks were inoculated into four cultural media, CMA, MEA, MRBA and PDA, with 4 blocks per plate, the inoculation medium was placed in a 28 °C incubator [36].

The soil fungi were separated using the dilution plating technique [37,42]. Ten grams of soil was added to a 250 mL conical flask containing 90 mL of sterile water in rotation and mixed at 160 rpm for 25 min. Subsequently, serial 10-fold dilutions were performed, and 100 µL aliquots of 10^−3^ and 10^−4^ dilutions of each sample were plated for isolation of fungi by three culture media CZA, MRBA and PDA [37]. All plates were incubated at 28 °C for 3 days to observe and purify the fungi [37].

The fungi were purified by transferring a small amount of mycelium from the edge of each colony to a new PDA plate. Each pure strain was preserved on PDA media in two centrifuge tubes at room temperature.

### 2.4. Fungal Strain Identification

The total genomic DNA of each strain was extracted using the T5 direct PCR kit (TSE011; TsingKe, Beijing, China). Using the genomic DNA as the template, the internal transcribed spacer (ITS) regions spanning from the end of the 18S rDNA to the beginning of the 28S rDNA for each strain were amplified using the primer pair V9G/ITS4 [43]. Thermal cycling conditions for PCR amplification were set as follows: 98 °C for 5 min, 35 cycles of 98 °C for 10 s, 60 °C for 30 s, 72 °C for 25 s and a final extension at 72 °C for 5 min [44]. PCR products were sent to GENEWIZ, Inc. (Suzhou, China) for sequencing. The 1560 sequences were run through the BLASTN search page using the Megablast program (National Center for Biotechnology Information; Bethesda, MD, USA), where hits with more than 98% similarity with published papers and their accession numbers were obtained. The taxon names of BLAST search results were checked on the web of Fungal Names (https://nmdc.cn/fungalnames, accessed on 6 April 2023) and Catalogue of Life (https://www.catalogueoflife.org/data/taxon, accessed on 6 August 2023) [45], and the current names were used in the resulting analysis. According to Lücking et al. [46], the non-taxonomic term “fungi”, without italics or capitalization, was used to encompass Fungi, Oomycota and several other unrelated but fungus-like organisms. A total of more than 1000 *Trichoderma* strains were isolated, and those from the CT and TF treatments were selected for sequencing with at least one *Trichoderma* strain each from the leaf, stem and root of each plant and their corresponding rhizosphere soil samples, while all of those from CC and CF treatments were sequenced. All of the strains were identified to the genera level and the species name through BLAST as reference species names.

### 2.5. Data Analysis

The rarefaction curve was constructed using the iNEXT package ver.3.0.0, comparisons of the estimated and extrapolated species richness in plots were calculated based on 50 bootstrap replicates at a 95% confidence interval [47]. All of the results except the effects of culture medium on *Trichoderma* and *Fusarium* with high isolation efficiency were analyzed according to 1275 strains. A Krona pie chart of 1275 strains was generated using the OmicShare Tools (http://www.omicshare.com/tools/, accessed on 6 August 2023), while 1584 strains of *Trichoderma harzianum* from the CT and TF treatments and 154 strains of *Fusarium oxysporum* from the CF and TF treatments were not included in the Krona pie chart. The construction of Venn diagrams and all statistical analyses of 1275 strains were performed using imageGP (http://www.bic.ac.cn/imageGP/, accessed on 6 August 2023) [48]. The diversity of fungi was analyzed with the Shannon index [49], and the number of genera and Simpson index were calculated to determine the homogeneity of fungi [50]. Significance analysis of the rhizosphere and endophytic fungal genera, Shannon index and Simpson index was performed by taking each period in the field and greenhouse as a replicate, with a total of 4 replicates per medium, and counting the number of genera, Shannon index and Simpson index. Then, the number of genera, Shannon index and Simpson index were compared using ANOVA and two-way ANOVA followed by Bonferroni correction (multiple comparisons) [51] and a Wilcoxon matched pairs test (pairwise comparisons) [52].

## 3. Results

### 3.1. Community Composition of Endophytic Fungi and Rhizosphere Soil Fungi

**Figure 1:**
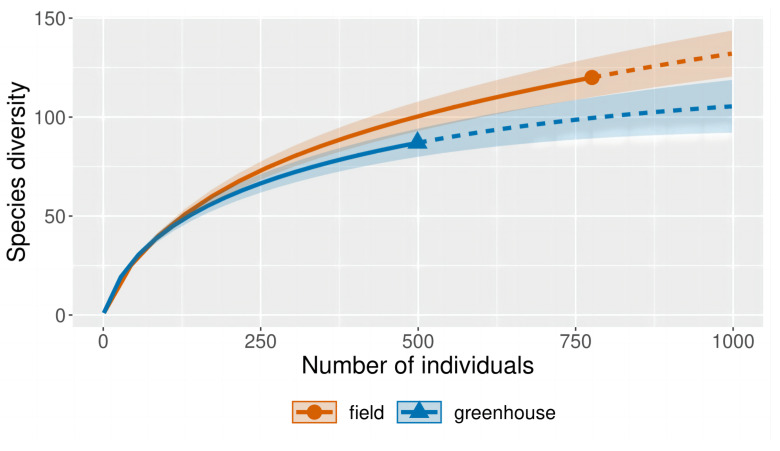
Rarefaction curves with 95% confidence intervals of estimated species richness of the isolated fungal community of cucumber in the field and greenhouse. The rarefaction curve is represented by the solid line, while the extrapolation curve is represented by the dashed line.

A total of 1275 strains, specifically 1273 strains of kingdom Fungi and 2 strains of kingdom Chromista isolated from cucumber plant tissues samples and rhizosphere soil, were analyzed. With the isolation depth near the saturation point of the sparse curve, the sampling effort was sufficient to achieve the diversity of the isolated strains (Figure 1).

**Figure 2:**
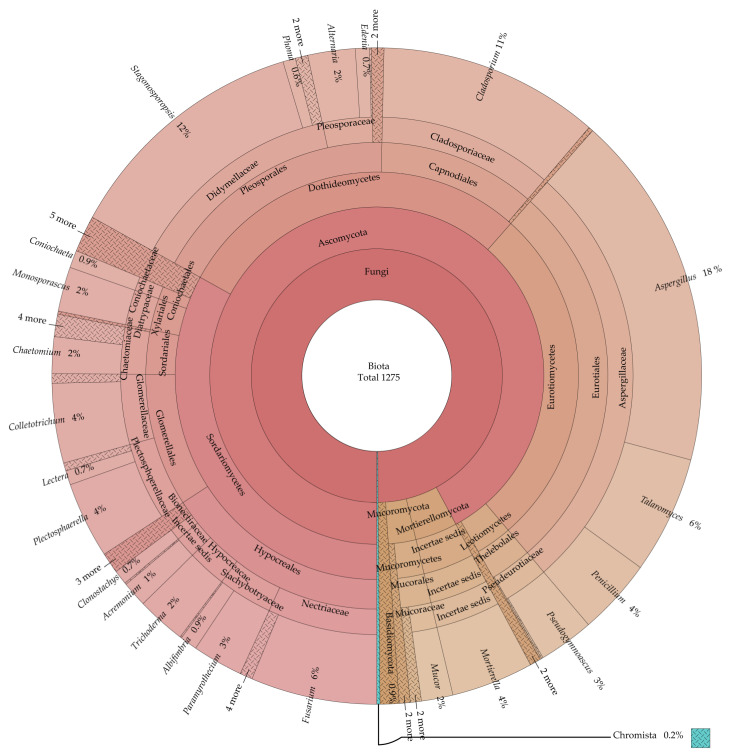
Krona diagram of all isolated fungi.

Through rDNA ITS sequence analysis, all 1273 strains were classified into 72 genera in kingdom Fungi, which include three phyla, 10 classes, 24 orders, 40 families (Table S1). As shown in Figure 2, the percentage of Ascomycota was highest, followed by Mucoromycota and Basidiomycota. In Ascomycota, Sordariomycetes made up the greatest proportion of the class, followed by Dothideomycetes and Eurotiomycetes. With regard to orders, Eurotiales, Pleosporales and Hypocreales were the most abundant. On a familial level, Aspergillaceae, Didymellaceae and Cladosporiaceae were the most abundant. Among all isolated fungi, *Aspergillus*, *Stagonosporopsis* and *Cladosporium* species were the top three genera, and they also accounted for the largest proportion of Ascomycetes. Among Mucoromycota phylum, the genus *Mucor* was the most isolated with 20 strains, followed by *Actinomortierella* and *Cunninghamella* with 4 strains, *Rhizopus* with 3 strains, *Umbelopsis* with 2 strains and *Linnemannia* with 1 strain (Table S1). In Mortierellomycota phylum, the genus *Mortierella* was isolated with 52 strains. In Basidiomycota phylum, a total of seven genera, *Malassezia* (three strains), *Rhizoctonia* (three strains), *Ceratobasidium* (two strains), *Irpex* (one strain), *Moesziomycesi* (one strain), *Schizophyllum* (one strain) and *Trametes* (one strain), were obtained (Table S1).

In addition, one strain from each of the genera *Globisporangium* and *Pythium* were also isolated, which belong to kingdom Chromista, phylum Oomycola, class Peronosporea, order Peronosporales and family Pythiaceae.

### 3.2. Influence of Culture Medium on Fungal Isolation

**Figure 3:**
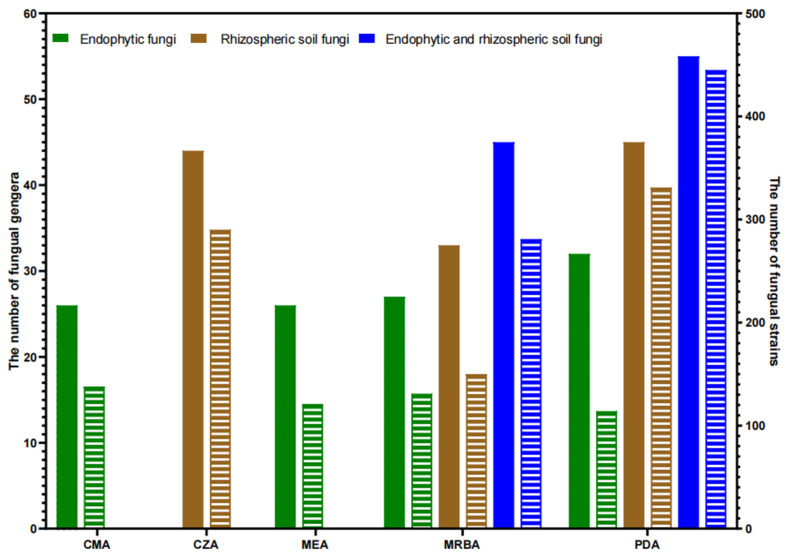
Comparison of the number of genera and strain number among different cultural media from cucumber plants and rhizosphere soil samples. The columns filled with colors indicate the number of fungal genera, while the columns filled with horizontal lines indicate the number of fungal strains.

**Figure 4:**
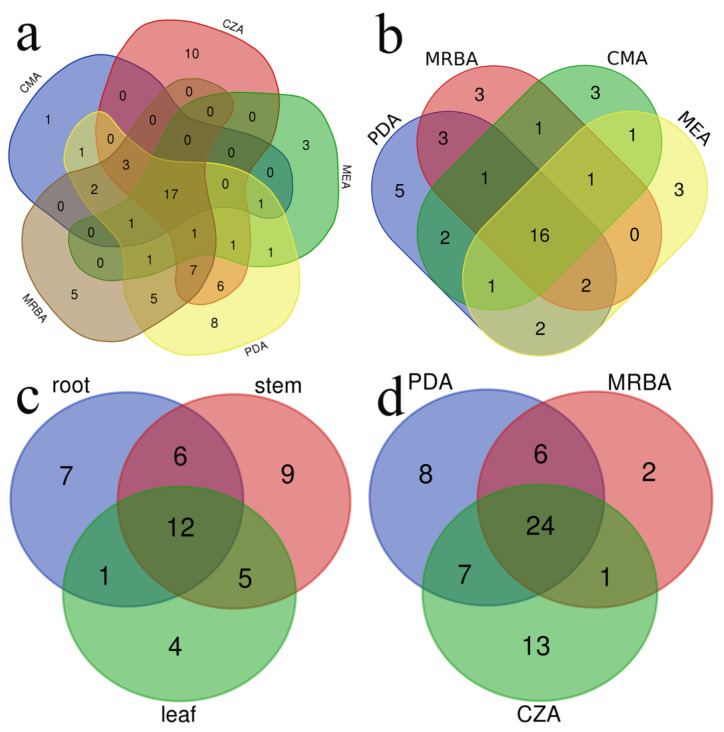
Venn diagram shows (**a**) genera of all isolated fungi with different cultural media; (**b**) genera of isolated endophytic fungi with different cultural media; (**c**) genera of isolated endophytic fungi in roots, stems and leaves; (**d**) genera of isolated soil fungi with different cultural media.

A total of five types of cultural media were utilized in this investigation, yielding a total of 74 fungal genera. Each culture medium yielded between 26 and 55 distinct fungal genera (Table 2, Figure 3). Furthermore, there were 28 fungal genera with high isolation efficiency. Among them, 17 fungal genera were successfully isolated using all five media, 16 and 24 genera were isolated with all four endophytic fungi isolation media and all three rhizosphere soil fungi isolation media individually and 12 genera were isolated from roots, stems and leaves of cucumber plants (Table 3; Figure 4a–d).

The number of genera, strains and endophytic genera of endophytic fungi and soil fungi isolated with each medium are listed in Table 2. Specifically, 10 genera of fungi were isolated only by CZA, which yielded the highest number of strains, while 8 genera were isolated only by PDA, 5 genera were isolated only by MRBA, 3 genera were isolated only by MEA and 1 genus was isolated only by CMA (Table 2 and Table S1). In endophytic fungi, the number of genera isolated by PDA was the highest, while the number of strains isolated by PDA was lower than those by CMA. PDA yielded the highest number of genera and strains in the rhizosphere soil fungi (Figure 3).

If only one medium is selected, PDA yields the highest number of genera isolated from cucumber endophytes, rhizosphere soil fungi and both compared to other media.

PDA alone isolated endophytic fungi of 32 genera accounting for 72.7% of all four media isolated to 44 genera, rhizosphere soil fungi of 45 genera accounting for 73.8% of all three media isolated to 61 genera and both endophytic and rhizosphere soil fungi of 55 genera accounting for 74.3% of all five media isolated to 74 genera (Table 2). There were 22 genera of endophytic fungi isolated with both PDA and MRBA, accounting for 81.5% of all endophytic fungi isolated with MRBA. Only five genera were isolated with MRBA but not PDA, accounting for 11.4% of all endophytic fungal genera. There were 30 genera of rhizosphere soil fungi isolated with both PDA and MRBA, accounting for 90.9% of all rhizosphere soil fungi isolated with MRBA. Only three were confirmed to be isolated with MRBA and not with PDA, accounting for 4.9% of all rhizosphere soil fungal genera.

### 3.3. Effects of Culture Medium on Endophytic Fungal Diversity

**Figure 5:**
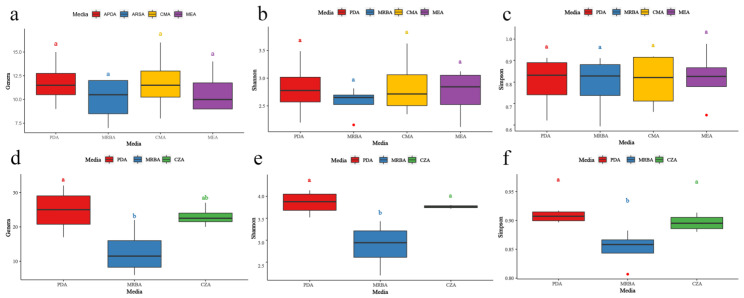
Diversity indices of fungi isolated with different cultural media. (**a**–**c**): The number of genera, Shannon index and Simpson index of endophytic fungi isolated on different cultural media; (**d**–**f**): the number of genera, Shannon index and Simpson index of soil fungi isolated on different cultural media; box graph data are all mean ± s.d. (*n* = 4); a, b = the same letter indicates no significant difference of *p* < 0.05.

In the isolation of endophytic fungi, there were no significant differences among the four media (CMA, MEA, MRBA and PDA). The number of genera isolated with each medium was 11.75 ± 2.50, 10.00 ± 2.44, 11.75 ± 3.30 and 10.75 ± 2.36 (Figure 5a), and the Shannon index was 2.81 ± 0.53, 2.57 ± 0.29, 2.85 ± 0.56 and 2.73 ± 0.45 (Figure 5b), respectively; Simpson’s index was 0.80 ± 0.13, 0.79 ± 0.14, 0.81 ± 0.13 and 0.82 ± 0.14 (Figure 5c), respectively. The number of endophytic genera and Shannon index of fungi isolated with PDA was larger than those of the other media.

A total of 504 strains of endophytic fungi were isolated by PDA, MRBA, MEA and CMA media, belonging to 21 orders, 33 families and 44 genera (Figure 4b). The highest number of genera was recovered from PDA with 131 strains and 32 genera, followed by MRBA (114 strains, 27 genera), CMA (138 strains, 26 genera) and MEA (121 strains, 26 genera) (Table 2). There were 16 genera of endophytic fungi obtained in all four media (Table 3). Among the endophytic fungi, *Cladosporium, Colletotrichum* and *Stagonosporopsis* were the genera with the highest number of strains isolated from the four cultures. On the other hand, five, three, three and three genera were unique to the isolated endophytic fungi from the PDA, MRBA, CMA and MEA media, respectively (Figure 4b). The genera *Acremonium*, *Aureobasidium*, *Cephalotheca*, *Humicola* and *Pythium* were obtained only on PDA, while *Globisporangium*, *Harknessia* and *Trametes* were isolated only from MRBA; *Boeremia*, *Clonostachys* and *Moesziomyce* were isolated only from CMA; and *Ceratobasidium*, *Curvularia* and *Preussia* were isolated only from MEA.

There were 20 genera of endophytic fungi isolated with PDA and CMA (Figure 4b), accounting for 76.9% of all endophytic fungal genera isolated with CMA. Only six genera were isolated with CMA but not PDA, accounting for 13.6% of all endophytic fungal genera. There were 17 genera of endophytic fungi isolated with PDA and MEA, accounting for 65.4% of all endophytic fungi isolated with MEA. Only five of them were isolated with MEA but not PDA, accounting for 11.4% of all endophytic fungal genera. Compared with PDA, six, five and five genera of specific fungi were isolated with CMA, MEA and MRBA, respectively (Figure 4b).

### 3.4. Effect of the Medium Type on Endophytic Fungal Diversity in Different Tissues of the Plant

The most genera of fungi isolated with the four different cultural media (CMA, MEA, MRBA and PDA) were 112 strains (32 genera) in the stems, followed by 240 strains (26 genera) in the roots and 152 strains (22 genera) in the leaves (Figure 4c). The number of endophytic fungi isolated on different parts of the plant on different cultural media was remarkably different (two-way ANOVA test, *p* < 0.05). When MEA was adopted, the number of genera isolated from stems (6.25) was significantly greater than that from roots (2.50) (Table 4). In the roots, the number of endophytic fungi isolated by MRBA (6.50) was significantly higher than that isolated on MEA (2.50), but in the stems, the number of fungi isolated with MRBA was significantly lower than that isolated with PDA (Table 4).

MRBA was the medium with the largest number of root fungal genera isolated among the four media, significantly more than MEA. In roots, *Fusarium, Monosporascus* and *Stagonosporopsis* were the three genera with the highest number of strains obtained. The greatest number of *Monosporascus* and *Stagonosporopsis* strains were observed in MEA and PDA media, respectively. CMA yielded the highest number of *Fusarium* strains. MRBA isolated the largest number of *Monosporascus* strains. *Preussiacan* was only isolated from the root using MEA, and *Trametescan* strains were only isolated from the root using MRBA (Table S1).

In the stem, PDA showed significantly higher genera separation than MRBA did. In the stem, *Cladosporium, Colletotrichum* and *Stagonosporopsis* were the three genera with the highest number of strains obtained. In addition to MEA, *Stagonosporopsis* was the main isolated genus in the other three media. *Moesziomyces* was isolated from stems only by CMA. *Acremonium*, *Cephalotheca, Humicola* and *Pythium* were isolated from stems using only PDA. *Harknessia* was only isolated from the stem using MRBA (Table S1).

In the leaves, the number of fungal genera isolated by the four medium differences was not significant. In the leaves, *Alternaria, Cladosporium* and *Stagonosporopsis* strains were the three genera with the highest number of strains obtained. Among them, *Stagonosporopsis* strains had the most strains in all four cultures. *Clonostachys* strains were isolated only from leaves using CMA, *Curvularia* strains were isolated only in leaves using MEA and *Aureobasidium* strains were isolated from leaves only using PDA (Table S1).

### 3.5. Effects of Culture Medium on Soil Fungal Diversity

In rhizosphere soil fungi, there were significant differences in the number of genera, Shannon index and Simpson index of fungi isolated by CZA, MRBA and PDA. The number of genera, Shannon index and Simpson index of soil fungi isolated with CZA, MRBA and PDA cultural media were 22.75 ± 3.10, 11.75 ± 7.41, 24.50 ± 6.45 (Figure 5d); 3.76 ± 0.04, 2.88 ± 0.53, 3.85 ± 0.28 (Figure 5e) and 0.90 ± 0.01, 0.85 ± 0.03, 0.91 ± 0.01 (Figure 5f), respectively. The Simpson’s index of soil fungi isolated by PDA was significantly higher than that of soil fungi isolated by MRBA (Figure 5d,f). The Shannon index of PDA and CZA was significantly higher than that of MRBA (Figure 5e). The diversity of soil fungi isolated on PDA was significantly higher than that from MRBA.

A total of 771 soil fungi were isolated by CZA, PDA and MRBA, belonging to 21 orders, 37 families and 61 genera (Figure 2, Table S1). There were 24 genera of fungi that could be isolated with all three cultural media, and the genus with the largest number of fungal strains isolated in all three cultural media were *Aspergillus* (Table 3). The number of soil fungi isolated with PDA was the highest (331 strains, 45 genera), followed by CZA (290 strains, 45 genera) and MRBA (150 strains, 33 genera) (Table 2). On the other hand, there were 13 genera, 8 genera and 2 unique genera of soil fungi isolated with CZA, MRBA and PDA media, respectively (Figure 4d). *Chaetomidium* and *Irpex* genera strains were obtained only in MRBA isolation.

There were 31 genera in the rhizosphere soil fungi isolated on PDA and CZA, accounting for 68.9% of all the fungal genera isolated with CZA. Fourteen genera were isolated with CZA but not PDA, accounting for 23% of all fungal genera isolated from rhizosphere soil.

### 3.6. Effects of Culture Medium on Trichoderma and Fusarium with High Isolation Efficiency

All 1617 *Trichoderma* strains (31 strains of *Trichoderma* spp. from CC and CF treatment, and 1586 strains of *T. harzianum* from CT and TF treatments) and 232 *Fusarium* strains (80 strains of *Fusarium* spp. from CC and CT treatment, and 152 strains of *F. oxysporum* from CF and TF treatments) isolated in this study were analyzed. In endophytic *Trichoderma* strains, significantly more strains were isolated with MEA than from MRBA, and the strain number was in the order MEA > CMA > PDA > MRBA (Table 5). In rhizosphere soil *Trichoderma* strains, there was no significant difference in the number of *Trichoderma* strains isolated with the three media, but the number of strains isolated with MRBA was the highest (514 strains), and CZA was the lowest (374 strains) (Table 5 and Table S1). The number of *Trichoderma* strains isolated with the three rhizosphere soil culture media was in the order of MRBA > PDA > CZA. There was no significant difference between *Fusarium* fungi isolated with each medium in endophytic and rhizosphere soil (Table 5). In the endophytic fungi, *Fusarium* strains were the most isolated by CMA, with a total of 40 strains, and PDA was the least, with a total of 26 strains. The number of endophytic *Fusarium* strains was in the order of CMA > MRBA > MEA > PDA. In the rhizosphere soil fungi, PDA had the largest number of *Fusarium* strains (44 strains), and MRBA isolated the fewest *Fusarium* strains (23 strains). The number of rhizosphere soil *Fusarium* strains obtained by the isolation medium was PDA > CZA > MRBA.

## 4. Discussion

Our study isolated a total of 72 fungal genera and 2 genera of oomycetes from leaves, stems and roots of cucumber plants and from cucumber rhizosphere soils. Among 1275 strains, the percentage of Ascomycota was highest, followed by Mucoromycota and Basidiomycota in kingdom Fungi, and fungus-like taxon names are based on the current names in phyla of Oomycota in kingdom Chromista [46]. *Aspergillus*, *Stagonosporopsis* and *Cladosporium* were the top three genera, and they also accounted for the largest proportion of Ascomycetes (Figure 2). Among the five cultural media tested, PDA yielded the highest number of fungal genera from both cucumber endophytic and rhizosphere soil samples. All five media were able to isolate unique genera. It should be noted that CZA and PDA can isolate as many as 10 and 8 unique genera in total. Significant differences among the four cultural media for the isolation of cucumber endophytic fungi and the three cultural media for rhizosphere fungi were also tested. The 12 fungal genera were isolated both from four endophytic fungi culture media and three rhizosphere soil fungi culture media.

To obtain the highest diversity of endophytic fungi is one of the problems in the study of endophytic fungi. Among the cultural media used in the isolation of endophytic fungi, PDA is the most common medium [4,5,6,7,8,14,15,16,18,19,20,21,22], while modified PDA [9,17], MEA [4,5,10,19,20], CMA [7,11,13,20] and rose bengal have been added to the cultural medium MEA to inhibit fast growing fungi [20]; the studies mentioned above mostly used only one media or did not compare the isolation rate among several cultural media. However, for reports where the expression of endophytic fungi of *Digitaria bicornis* was high (1326) on PDA followed by MEA (1047) and MB (moist blotters, 643) [5], the isolation rate of endophytic fungi from *Broussonetia papyrifera* varied with culture media, and PDA (75.94%, 17 genera) produced the highest number of strains, followed by CZA (15.51%, 4 genera), SDA (4.81%, 7 genera) and GA (3.74%, 3 genera) [22], and PDA and YM media showed similar results for isolation of the endophytic fungi from *Oxalis acetosella* L., whereas CMA performed worse but still better than any of the TWA-media [7], showing that PDA is a better cultural media. MRBA and PDA had higher Shannon-Wiener and Simpson indices, indicating a better balance and diversity of endophytic fungi on MRBA and PDA. The Shannon-Wiener index and Simpson index of MEA were the lowest, indicating that MEA had high selectivity for fungi and was suitable for the isolation and culture of fungi of specific species.

Various cultural media have been employed for the isolation of rhizosphere fungi, and 3–4 different cultural media were often used [23,24,25,26,30,31,32], while two cultural media MEA and PDA [28] and only one [27,33] were also reported. Because of the growth of certain fungal species only on CZA or PDA, the use of more than one culture medium with a specific carbon source for the isolation of mycoflora from an ecological location was suggested by Vasanthakumari et al. [26]. PDA, commonly employed as a cultural medium for rhizosphere fungi isolation, is often combined with other cultural media to enhance isolation efficacy [24,25,26,28,30]. CZA was also used for isolation rhizosphere soil fungi of forest, grass and desert [24,26,32]. MRBA is frequently employed for the isolation of rhizosphere soil materials abundant in fungus due to its inclusion of rose bengal, which exhibits inhibition of bacterial growth and expansion of fungal colonies properties [25,41]. There was no comparison of isolation efficiency with different cultural media in most of the reports. However, in comparison to cultural media CZA and WA, PDA was reported to support a large number of fungal species, suggesting its superiority as an isolation medium [26]. When complex media (TSA and Martin) were used, the numbers of fungi in all three (sorghum, eucalyptus and forest) soils were higher compared to those in saline media (Thorton and Czapeck) [31]. MEA is also a commonly used cultural media for the isolation of rhizosphere fungi from *Bauhinia*, mangroves, peat and canyon soils [23,28,29,30,32]. However, in this study, the MEA was not selected because the inoculant *Trichoderma harzianum* grew so fast on MEA and covered other fungi. In this study, among the three selected rhizosphere soil fungi isolation media, the PDA (45 genera and 331 strains) isolation effect was significantly better than MRBA (33 genera and 150 strains). This result is different from Vieira et al. [31], whose view is that complex media containing peptides, amino acids and vitamins have a better isolation effect than simple saline media. In this study, 59 genera and 621 fungal strains were isolated with two cultural media of CZA and PDA (accounting for 96.7% of the total 61 genera and 80.5% of the total 771 strains), while 53 genera and 440 rhizosphere fungal strains with CZA and MRBA (accounting for 86.9% genera and 57.1% strain number), 48 genera and 481 rhizosphere fungi strains were isolated with MRBA and PDA (accounting for 78.7% genera and 62.5% strain number). When all three media were used, only three genera and strains were increased compared to the two cultural media CZA and PDA. It is speculated that rose bengal contained in MRBA may inhibit the growth of fungi. According to our study, if only one cultural media was used for isolation of rhizosphere foil fungi, PDA is the best, then CZA, and MRBA is the worst. The combination of two media, CZA and PDA, was superior to the other two media combinations, and three cultural media including MRBA was beneficial for specific genera *Chaetomidium*, *Irpex* and *Monosporascus*.

Therefore, PDA is a suitable medium for the simultaneous isolation of both cucumber endophytic and rhizosphere soil fungi. Study of the plant endophytic or rhizosphere soil fungal diversity with different cultural media has been reported, but research involving both plant endophytic and rhizosphere soil fungal diversity with different cultural media is rarely reported. In this study, for the endophytic *Trichoderma* spp., the fungal strain number isolated on MEA was significantly higher than that from MRBA. It was reported that *Pyricularia grisea* as an endophyte in *Digitaria bicornis* was expressed only on MEA [5], and in this study only one strain of *Pyricularia* sp. was isolated from the rhizosphere with CZA. Certain fungal species from rhizosphere soil were also only reported for CZA or PDA [26], so the genera specific to each cultural media resulted from the samples and isolation conditions in this study.

Among 12–16 endophytic fungal genera with high isolation efficiency, *Alternaria* [53], *Aspergillus* [53], *Chaetomium* [14,53], *Cladosporium* [53], *Colletotrichum* [14], *Fusarium* [14,53,54], *Plectosphaerella* [55] and *Talaromyces* [56] were found to be isolated from leaf, stem and root of cucumber plants, and most reports were from our lab [14,53,54,55,56]. The identification method of Yan et al. was according to the morphological characteristics of the fungi [54]. So, some cucumber endophytic fungal genera are stable from samples of different years and difference places. Besides the genera mentioned above, *Apiospora* and *Nigrospora* were isolated from samples of open cultivation with three cultural media and *Bipolaris* and *Penicillium* from samples of greenhouse cultivation with two cultural media [53]. In this study, *Apiospora* as rhizosphere fungi and *Penicillium* as endophytic and rhizosphere fungi were found to have high isolation efficiency, while the fungal genera *Bipolaris* and *Nigrospora* were not found in this study. Different cucumber varieties and geographical conditions might influence the diversity of endophytic fungi in cucurbit plants [14,53].

In 24 genera from all three media used to isolate rhizosphere fungi, *Fusarium* [57,58], *Mortierella* [57], *Mucor* [58], *Penicillium* [57,58] and *Trichoderma* [57,58] were reported to be isolated common or dominant groups in the cucumber rhizosphere soils, and the fungal genera were determined by morphological observation [57,58]. Nine genera, *Coniothyrium*, *Cylindrocarpon*, *Gliocladium*, *Monocillium*, *Myrothecium*, *Paecilomyces*, *Phialocephala*, *Staphylotrichum* and *Verticillium*, were isolated of total 13 genera common in the four treatments in the study of the effects of *Pseudomonas* biocontrol inoculants on nontarget rhizosphere fungi [57], which were not found in this study. The populations of *Pencillium*, *Fusarium* and *Rhizopus* were significantly lower in the two resistant cucumber cultivars than those in the two cultivars susceptible to cucumber fusarium wilt (*Fusarium oxysporum*, K1 and K2) [58]. The different geographical conditions of Zürich, Switzerland [57]; Harbin, China [58]; and Beijing, China (this study), may have impacted the diversity of culturable fungi in the cucumber rhizosphere. Those fungal genera isolated as cucumber endophytes and from rhizosphere soil samples are worth further research.

## 5. Conclusions

In this study, we investigated the influence of the medium type on the isolation of cucumber endophytic fungi and rhizosphere soil fungi. We found that the type of medium did not have a significant effect on the isolation of endophytic fungi, but the number of rhizosphere soil fungal genera isolated with PDA was significantly higher than that from MRBA. Based on the data obtained here, we suggest that PDA can be used as a basic medium for the isolation of both cucumber endophytic and rhizosphere soil fungi, and different media can be considered for the isolation of endophytic fungi from different parts of the plant and to emphasize specific fungal genera.

## Figures and Tables

**Table 1:** Details of collected cucumber plants and their corresponding rhizosphere soil.

Collection Time	Location	Growth Stage [38]	Number of Plants	Soil Number	Treatment Row/Pot Number
20 July 2022	Field	Seedling	16	16	CC ^1^-4; CT-4; CF-4; TF-4
5 August 2022	Greenhouse	Seedling	16	16	
14 August 2022	Field	Flowering	16	16	
21 August 2022	Greenhouse	Vine growth	16	16	
		Total	64	64	

^1^ CC: control; CT: inoculation with *Trichoderma harzianum*; CF: inoculation with *Fusarium oxysporum* f. sp. *cucumerinum*; TF: inoculation with *T. harzianum* and *F. oxysporum* f. sp. *cucumerinum*.

**Table 2:** Fungal genera isolated by each medium.

Fungal Type	Media	Number of Genera	Strain Number	Specific Genera Compared to PDA/Specific Genera with PDA
Endophytic fungi (Total 44 genera, 504 strains)	CMA	26	138	*Acrocalymma *^1^; *Boeremia*; *Clonostachys*; *Graphium*; *Moesziomyces *^2^; *Mucor*; *Paramyrothecium*
	MEA	26	121	*Ceratobasidium*; *Curvularia*; *Mucor*; *Paramyrothecium*; *Preussia*
Rhizosphere soil fungi (Total 61 genera, 771 strains)	CZA	45	290	*Antarctomyces*; *Bisifusarium*; *Edenia*; *Geomyces*; *Marquandomyces*; *Meyerozyma*; *Microascus*; *Monosporascus*; *Nalanthamala*; *Ochroconis*; *Pyricularia*; *Sarocladium*; *Striaticonidium*; *Trichocladium*
Endophytic fungi Rhizosphere soil fungi Total fungi (74 genera, 1275 strains)	PDA	32	131	*Acremonium*; *Aureobasidium*; *Cephalotheca*; *Humicola*; *Pythium*
		45	331	*Boeremia*; *Cephalotheca*; *Engyodontium*; *Glomerella*; *Linnemannia*; *Metarhizium*; *Paraisaria*; *Schizophyllum*
		55	445	
Endophytic fungi	MRBA	27	114	*Globisporangium*; *Graphium*; *Harknessia*; *Mucor*; *Trametes*
Rhizosphere soil fungi		33	150	*Chaetomidium*; *Irpex*; *Monosporascus*
Total fungi		45	281	

^1^ the only italic fonts indicate the specific genera compared to PDA or only from PDA as endophytic fungi/rhizosphere soil fungi compared to other cultural media; ^2^ Bold, underline and italic fonts indicate the specific genera isolated only by this media.

**Table 3:** Fungal genera with high isolation efficiency.

	Genera	Isolated from All 5 Cultural Media	Isolated from All 3 Media Used to Isolate Rhizosphere Fungi	Isolated from All 4 Media Used to Isolate Endophytic Fungi	Isolated from Roots, Stems and Leaves of Cucumber Plants
1	*Acremonium*		+		
2	*Acrocalymma*			+	
3	*Albifimbria*		+		
4	*Alternaria* *	+	+	+	+
5	*Apiospora*		+		
6	*Aspergillus*	+	+	+	+
7	*Chaetomium*	+	+	+	
8	*Cladosporium*	+	+	+	+
9	*Clonostachys*		+		
10	*Colletotrichum*	+	+	+	+
11	*Coniochaeta*		+		+
12	*Edenia*	+		+	
13	*Fusarium*	+	+	+	+
14	*Humicola*		+		
15	*Lectera*		+		
16	*Malassezia*		+		
17	*Microdochium*	+		+	
18	*Monosporascus*	+		+	+
19	*Mortierella*		+		
20	*Mucor*	+	+		
21	*Paramyrothecium*	+	+		
22	*Penicillium*	+	+	+	+
23	*Phoma*		+		
24	*Plectosphaerella*	+	+	+	+
25	*Pseudogymnoascus*	+	+	+	
26	*Stagonosporopsis*	+	+	+	+
27	*Talaromyces*	+	+	+	+
28	*Trichoderma*	+	+	+	+
	Total	17	24	16	12

* Bold fonts indicate the fungal genera isolated as cucumber endophytes and from rhizosphere soil samples.

**Table 4:** The number of genera of endophytic fungi isolated in different plant parts with each medium.

Plant Part/Cultural Medium	CMA	MEA	PDA	MRBA
Root	5.50 a AB ^1^	2.50 b B	3.00 b AB	6.50 a A
Stem	7.75 a AB	6.25 a AB	8.75 a B	4.50 a A
Leaf	4.25 a A	3.75 ab A	4.75 b A	3.25 a A

^1^ The same row marked with different lowercase letters indicates significant differences between groups, and the same row marked with different capital letters indicates significant differences between groups.

**Table 5:** The isolate numbers of *Fusarium* and *Trichoderma* isolated as cucumber endophytes and from rhizosphere soil samples.

Media/Isolates	CMA	CZA	MEA	MRBA	PDA
*Fusarium* (E)^1^	10.00 ± 1.73 a ^3^	-	8.50 ± 4.04 a	9.25 ± 5.80 a	6.50 ± 3.70 a
*Fusarium* (R)^2^	-	7.00 ± 3.37 ab	-	5.75 ± 4.78 ab	11.00 ± 7.35 ab
*Trichoderma* (E)^1^	21.21 ± 3.69 ab	-	24.50 ± 4.65 a	15.75 ± 5.25 b	20.75 ± 5.32 ab
*Trichoderma* (R)^2^	-	93.50 ± 8.06 a	-	128.50 ± 53.98 a	100.00 ± 34.69 a

(E)^1^ indicate the isolates of the genera isolated as cucumber endophytes; (R)^2^ indicates the isolates of the genera isolated from rhizosphere soil samples. ^3^ The same row marked with different letters indicates significant differences between groups.

## Data Availability

Data is contained within the article or Supplementary Materials.

## Supplementary Materials

The following supporting information can be downloaded at: https://www.mdpi.com/article/10.3390/jof9090934/s1, Table S1: Strains isolate by different cultural media in this study including the accession number in GenBank. References [59,60,61,62,63,64,65,66,67,68,69,70,71,72,73,74,75,76,77,78,79,80,81,82,83,84,85,86,87,88,89,90,91,92,93,94,95,96,97,98,99,100,101,102,103,104,105,106,107,108,109,110,111,112,113,114,115,116,117,118,119,120,121,122,123,124,125,126,127,128,129,130] are cited in the Supplementary Materials.
